# Editorial Notes

**Published:** 1935

**Authors:** 


### Editorial Notes

**Death of Mr. Edward Robinson.**

In the death of Mr. Edward Robinson a great figure in Bristol life has passed away. He was one of the quiet, deeply religious, honourable and honoured men who have for many generations stood out as typical Bristol citizens. Like many other business heads in our city he gave no visible sign of "hustle." Probably he was not indeed a hustler, but he knew the value of unremitting attention to the work in hand, he was quick to grasp at new inventions or new ideas, and, like all the Robinson family, he knew the value of games in due season.

He had served his City well and faithfully, not only in his own business but in many public capacities, for he had been Lord Mayor in 1908 and an Alderman since 1924, President of the Anchor Society in 1887, President of the University Colston Research Society, a member of the University Council, Treasurer of the Bristol Baptist College, and for many years an active Justice of the Peace.

As President of the Queen Victoria Jubilee Convalescent Home Mr. Robinson had rendered invaluable service to the sick of Bristol, more especially to patients discharged convalescent from our great hospitals.

Although he was most careful that the Convalescent Home should keep the expenses within its income, he was always disappointed if the whole income was not spent. Yet there was generally a small sum put aside for contingencies so that extraordinary expenditure could be met or necessary extensions carried out. As President of the Home he was in constant touch with the institution and its inmates. His kindness and courtesy towards the administrative, medical and nursing staff were unvarying and as a Chairman of Committee he was businesslike and gracious.

### Meetings of Societies

